# The Effect of Cranial Nerve Stimulation on Swallowing: A Systematic Review

**DOI:** 10.1007/s00455-020-10126-x

**Published:** 2020-05-14

**Authors:** Michelle G. M. H. Florie, Walmari Pilz, Remco H. Dijkman, Bernd Kremer, Anke Wiersma, Bjorn Winkens, Laura W. J. Baijens

**Affiliations:** 1 Department of Otorhinolaryngology, Head and Neck Surgery, Maastricht University Medical Center, PO Box 5800, 6202 AZ Maastricht, The Netherlands; 2 GROW-School for Oncology and Developmental Biology, Maastricht University Medical Center, Maastricht, The Netherlands; 3 School for Mental Health and Neuroscience – MHeNs, Maastricht University Medical Center, Maastricht, The Netherlands; 4 Department of Methodology and Statistics, CAPHRI - Care and Public Health Research Institute, Maastricht University, PO Box 616, 6200 MD Maastricht, The Netherlands

**Keywords:** Dysphagia, Deglutition disorder, Swallowing, Cranial nerve, Cranial nerve stimulation

## Abstract

This systematic review summarizes published studies on the effect of cranial nerve stimulation (CNS) on swallowing and determines the level of evidence of the included studies to guide the development of future research on new treatment strategies for oropharyngeal dysphagia (OD) using CNS. Studies published between January 1990 and October 2019 were found via a systematic comprehensive electronic database search using PubMed, Embase, and the Cochrane Library. Two independent reviewers screened all articles based on the title and abstract using strict inclusion criteria. They independently screened the full text of this initial set of articles. The level of evidence of the included studies was assessed independently by the two reviewers using the A–B–C rating scale. In total, 3267 articles were found in the databases. In the majority of these studies, CNS was used for treatment-resistant depression or intractable epilepsy. Finally, twenty-eight studies were included; seven studies on treatment of depression, thirteen on epilepsy, and eight on heterogeneous indications. Of these, eight studies reported the effects of CNS on swallowing and in 20 studies the swallowing outcome was described as an adverse reaction. A meta-analysis could not be carried out due to the poor methodological quality and heterogeneity of study designs of the included studies. These preliminary data suggest that specific well-indicated CNS might be effective in reducing OD symptoms in selective patient groups. But it is much too early for conclusive statements on this topic. In conclusion, the results of these studies are encouraging for future research on CNS for OD. However, randomized, double-blind, sham-controlled clinical trials with sufficiently large sample sizes are necessary.

## Introduction

Cranial nerves play an important role in swallowing, a complex cognitive, sensorimotor process of moving any bolus from the mouth to the stomach [1, 2]. The stages of swallowing can be seen as a complex activity along a spectrum of automaticity, with the esophageal stage being most automatic and the oral stage the least [1]. The nervous system controlling swallowing movements has mammalian brain stem and midbrain patterning control systems (automatic areas) as well as cortical and subcortical volitional areas [2]. But in all stages of swallowing the cranial nerves play a major role in modulating swallowing execution and their integrity is indispensable [3].

The trigeminal nerve (TN), the fifth cranial nerve, controls somatosensation of the face and the anterior two-thirds of the tongue [4, 5]. It provides motor innervation of (1) the mylohyoid muscle and the anterior belly of the digastric muscle, which are hyolaryngeal elevators; (2) the masticatory muscles, such as the masseter, temporalis, medial and lateral pterygoid muscles; and (3) the tensor veli palatini muscle. Impairment of the TN can cause problems in the preparatory oral and oral phase of swallowing due to poor mastication and poor stabilization of the mouth floor. TN dysfunction will also result in a decreased hyolaryngeal excursion during the pharyngeal phase of swallowing due to mylohyoid and anterior belly digastric muscle impairment.

The facial nerve (FN), the seventh cranial nerve, conveys taste sensation of the anterior two-thirds of the tongue [4]. Furthermore, the FN controls motor movement of the muscles of facial expression such as the orbicularis oris and buccinator muscle, both playing a role in lip closure and prevention of oral residue. The FN also provides motor innervation of the stylohyoid muscle and the posterior belly of the digastric muscle. These muscles retract the hyoid bone posterosuperiorly and assist in glossopalatal closure. Innervation of the submandibular and sublingual salivary glands is provided by the FN too. Impairment of the FN can result in decreased taste perception, poor bolus formation during the preparatory oral phase, anterior bolus spilling, postswallow oral residue, and dry mouth.

The glossopharyngeal nerve (GN), the ninth cranial nerve, controls somatosensation of the posterior one-third of the tongue and of the mucosa of the soft palate and the upper pharyngeal tract [4]. The autonomic innervation of the parotid gland is also provided by the GN. The motor innervation of the GN is the innervation of the stylopharyngeus muscle, which is a laryngeal elevator assisting in the opening of the upper esophageal sphincter (UES). The GN also plays a key role in soft palate elevation as the salpingopharyngeus muscle is innervated by the GN and this muscle fuses inferiorly with the fibers of palatopharyngeus muscle. GN dysfunction can result in impaired pharyngeal bolus transport and impaired UES opening resulting in postswallow pharyngeal pooling.

The vagal nerve (VN), the tenth cranial nerve, provides both motor and sensory innervation, and plays an important role in the pharyngeal phase of swallowing [4, 6]. The motor fibers of the VN innervate all striated muscles of the larynx and pharynx, except the stylopharyngeus muscle and the tensor veli palatini muscle, which are innervated by the GN and by the TN, respectively. The pharyngeal branches of the VN innervate the levator veli palatini, salpingopharyngeus, palatopharyngeus, palatoglossus, and the uvular muscle. The external superior laryngeal nerve (SLN) supplies the motor innervation of the cricothyroid muscle. The recurrent laryngeal nerve (RLN) is responsible for the motor innervation of all intrinsic laryngeal muscles except for the cricothyroid muscle. Various branches of the VN, like the RLN and the internal branch of the SLN (ISLN), provide mucosal sensory innervation of the pharynx, larynx, and proximal trachea. Impairment of the VN can cause poor velopharyngeal seal and nasal reflux, weak pharyngeal contraction, reduced vocal fold adduction resulting in dysphonia and poor cough effectiveness, impaired UES opening—postswallow pharyngeal pooling, and silent aspiration.

The hypoglossal nerve (HN), the twelfth cranial nerve, innervates all intrinsic and extrinsic tongue muscles, except for the palatoglossus muscle (VN). The HN is a nerve with exclusively motor function controlling all movements of the tongue. Besides dysarthria, impairment of the HN can cause problems with oral control of the bolus, bolus propulsion due to poor lingual pressure and driving forces resulting in premature posterior spill of the bolus to the pharynx, postswallow oral residue, etc.

The ansa cervicalis (AC), the connection between the cervical plexus (C1, C2) and the HN, is a loop of nerves, which innervates the omohyoid muscle’s superior belly as well as the superior part of the sternothyroid and the sternohyoid muscles [3, 7]. Activation of these muscles initiates hyolaryngeal elevation. Furthermore, the AC also seems to assist in airway protection by compression of the quadrangular membrane thereby assisting in closure of the laryngeal inlet in collaboration with the TN and VN [8]. Function loss of the AC can cause an impaired UES opening, resulting in postswallow pharyngeal pooling.

Cranial nerve palsy is characterized by a decreased or complete loss of function of one or more cranial nerves. The etiology may be congenital or acquired. Multiple cranial neuropathies of cranial nerves involved in swallowing are common, particularly in lesions arising from tumors, trauma, head-and-neck surgery, impaired blood flow, and infections. For example, cranial nerves FN, VN, HN, and AC are especially at risk during head-and-neck cancer surgery [9]. Tumor extension into a cranial nerve often results in sacrificing this nerve during a radical or modified radical neck dissection. Also in thyroid gland surgery cranial nerves may be at risk in particular the VN–RLN. If multiple cranial nerve palsy occurs for no apparent reason, abnormalities in the base of the skull and brain should be considered. Also paraneoplastic symptoms or infections caused by neurotropic viruses such as those of the herpes group should be part of the differential diagnoses. Finally, cranial nerve palsy may be the result of a stroke [8]. Bilateral cranial nerve palsy or asymmetrically neuromuscular representation and the potential recovery depend on the size and site of the lesion in the brain.

During the past two decades, several studies on cranial nerve stimulation (CNS) have been published. CNS is a medical treatment for symptoms of various diseases, in which electrical signals stimulate cranial nerves in order to modulate the activity of targeted brain regions or to modulate the action of peripheral structures. Currently, there are two types of CNS that are used in daily clinical practice, namely vagal nerve stimulation (VNS) and hypoglossal nerve stimulation (HNS).

CNS has been applied for several diseases and/or syndromes such as epilepsy, obstructive sleep apnea syndrome, obesity, neuropsychiatric disorders (depression, obsessive compulsive disorder, panic disorder, pain disorder, post-traumatic stress disorder, etc.), and asthma exacerbations [10–20]. Few studies described the effect of CNS on swallowing and, consequently, the overall effect of this intervention on swallowing remains unclear.

Various treatments for oropharyngeal dysphagia (OD) have been described in the literature, including swallowing exercises that aim to change swallow physiology through targeting strength and/or range of movement of muscles and/or neuroplasticity in the brain, surgical interventions (UES interventions), bolus modification (modified texture diet), neuromuscular electrical stimulation (E-stimulation etc.), transcranial magnetic stimulation, postural and airway protective strategies/maneuvers that facilitate swallowing, pharmacological interventions, etc. [21–24]. CNS has only been scarcely investigated as a treatment for OD and evidence of its effectiveness is part of the subsequent systematic review in the present study. The systematic review was build based on the following question: Is there any scientific evidence that CNS can be used as a novel treatment for OD in specific patient populations?

The aim of this systematic literature review was to present an overview of the studies on the effect of CNS on swallowing and to determine the level of evidence of these studies to guide the development of future research on new treatment strategies for OD using CNS.

## Methods

### Identification and Selection of Studies

**Fig. 1:**
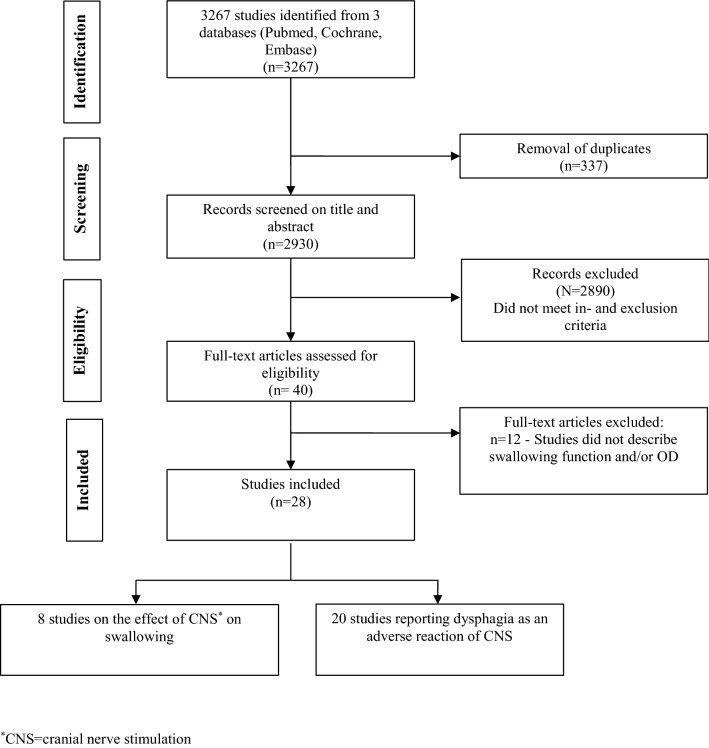
PRISMA study selection

This review was conducted following the Cochrane Collaboration criteria for systematic reviews [25]. The literature search using the electronic databases Embase, PubMed, and the Cochrane library was carried out on November 1, 2019 by two independent investigators. Search terms were related to dysphagia and to CNS. The search strategy is presented in Table 1. The search was limited to articles published between January 1990 and October 2019. Studies were included if they described any effect of CNS on the oropharyngeal swallowing physiology even if the effect was reported as an adverse reaction (AR). The in- and exclusion criteria are listed in Table 2. Peer-reviewed journal articles written in the English, German, Portuguese, Spanish, French, or Dutch language were included in the search. Studies involving experiments on animals were excluded. Articles were also excluded if swallowing outcomes were not presented in the results. Two independent reviewers performed the first selection by screening all articles based on title and abstract. During the next step they independently screened the full text of the selected set of articles. Finally, the reference lists of the selected articles were screened for additional literature. All studies reporting on swallowing and CNS and meeting the in- and exclusion criteria were included. The level of agreement between the two reviewers for eligibility after full-text screening was obtained using percentage of agreement. Figure 1 comprises a flow diagram showing the article selection according to Preferred Reporting Items for Systematic Reviews and Meta-Analyses (PRISMA) [26].

**Table 1:** Systematic syntax

*PubMed (MeSH and free-text terms)*
(("Deglutition Disorders"[Mesh] OR "Deglutition"[Mesh]) OR (deglut* OR dysphag* OR swallow*)) AND (("Vagus Nerve Stimulation"[Mesh] OR "Electrodes, Implanted"[Mesh] OR "Implantable Neurostimulators"[Mesh]) OR (nerve stim* OR electr*))
("Deglutition Disorders"[Mesh]) OR "Deglutition"[Mesh]) OR deglut*) OR dysphag*) OR swallow*) AND (("Electrodes, Implanted"[Mesh]) OR "Implantable Neurostimulators"[Mesh]) AND "Cranial Nerves"[Mesh]))
*Embase (thesaurus and free-text terms)*
(((swallow* OR deglutition OR dysphagia) AND (cranial nerve OR trigeminal nerve OR facial nerve OR glossopharyngeal nerve OR vagus nerve OR accessory nerve OR hypoglossal nerve)) AND (electrode implant OR nerve stimulation OR vagus nerve stimulation)
(Swallow* or dysphag*) AND (cranial nerve OR cranial nerve stimulation)
(Swallow* or dysphag*) AND vagal nerve stimulation
*Cochrane (free-text terms)*
Cranial nerve stimulation OR trigeminal nerve stimulation OR facial nerve stimulation OR glossopharyngeal nerve stimulation OR vagal nerve stimulation OR accessory nerve stimulation OR hypoglossal nerve stimulation

*Truncation of search terms to broaden the search

**Table 2:** In- and exclusion criteria

*Inclusion criteria*
Studies describing swallowing function and/or OD associated with CNS
Studies describing OD and/or aspiration even as an adverse reaction of CNS
Studies on human subjects
*Exclusion criteria*
Studies including other forms of stimulation such as transcutaneous, intrapharyngeal, or deep brain stimulation
Studies on CNS that did not report swallowing function or OD as outcome variable or adverse reaction
Animal studies

*CNS* cranial nerve stimulation, *OD* oropharyngeal dysphagia

### Data Analysis and Assessment of the Level of Evidence

The level of evidence of the included studies was assessed using the A–B–C rating scale by Siwek et al. [27]. This assessment was carried out by both reviewers independently, blinded to each other’s results. This design-specific tool was chosen because the methodological quality of the majority of the included studies was not sufficient to carry out a validated critical appraisal assessment. According to the A–B–C rating scale, level A refers to high-quality randomized controlled trials and meta-analyses, level B refers to well-designed, non-randomized clinical trials, and level C refers to consensus or expert opinion. The level of agreement between the two reviewers for the A–B–C rating scale was obtained using percentage of agreement.

Data extraction included the following variables: sample size, indication for stimulation, cranial nerve number, type of nerve stimulator, stimulation parameters, measurement tool and outcome parameters, authors’ conclusion, and the number of OD-related ARs.

## Results

### General Results and Level of Evidence

A total of 2930 articles were identified after removing duplicates using EndNote X7.5 (Clarivate Analytics, Philadelphia, Pennsylvania) (Fig. 1). The percentage of agreement between the two reviewers for the first selection based on title and abstract was 68%. If agreement was not reached based on the title and abstract, the full text of the article was screened. The percentage of agreement between the two reviewers for eligibility after full text screening was 100%. Finally, 28 articles were included for subsequent assessment of the level of evidence (Table 3). The level of agreement between the two reviewers using the A–B–C rating scale was 100%. All the included articles were written in English. All eight studies on the effect of CNS on swallowing (Table 3) met the criteria of level C—expert opinion. Thus, a meta-analysis of the included studies was not carried out as the studies did not have sufficient methodological quality to warrant doing so. More specific, the majority of the studies presented serious methodological shortcomings (e.g., no clear description of the subjects selection criteria; little or incomplete information about diagnostic tools and protocol; no interpretation of the test results). Subsequently, a narrative descriptive review of the systematically included studies was performed. Table 3 provides an overview of the eight studies describing the effect of CNS on swallowing. In this table, the sample size, indication for stimulation, cranial nerve number, type of nerve stimulator, stimulation parameters, measurement tool and outcome parameters, and authors’ conclusion are summarized.

**Table 3:** Effects of cranial nerve stimulation on swallowing

Refs	Number of subjects (N), gender (M:F), and indication for stimulation	Cranial nerve number, type of nerve stimulator	Stimulation parameters (stimulus duration; current intensity; pulse frequency; pulse width)	Measurement tool and outcome parameters on swallowing	Authors' conclusion
[29]	*N* = 7 Children with intractable epilepsy	VN (CI) NCP model 100, and electrodes	-Almost continuous stimulation (MAX): 120 s "on", 0.2 min "off" -Individual settings (IN USE): 7–30 s "on", 0.3–5.0 min "off" -Stimulator turned off (OFF) General settings: at the start intensity progressively increased from 0.25 mA to 1.25–2.0 mA; frequency 30 Hz; pulse width 500 µsec	-Videoradiography during barium swallow, using an aspiration scale 0–3 (normal passage of barium, penetration located on the epiglottis or supraglottic penetration, and glottic aspiration) -*N* = 2 showed an increased degree of aspiration during almost continuous stimulation (MAX)	Aspiration is a potentially serious AR of VNS. It could be prevented if the stimulator would have an electrical switch that the patient or caregiver could turn off, if necessary
[30]	*N* = 8 Children with pharmacoresistant epilepsy	Left VN Type of nerve stimulator not specified	-Stimulator turned off -Customary settings: duration 3–4 min; current 1.0–2.0 mA -Maximum tolerated stimulation: duration 3–4 min; maximum current of 3.0 mA	-Videoradiography during barium swallow to determine laryngeal penetration/aspiration during electrical stimulation. No further information on the protocol was provided -*N* = 1 showed worsening of OD from “normal” to “laryngeal penetration” during VNS at customary settings *N* = 2 showed worsening of the OD from “normal” to “laryngeal penetration” at maximum tolerated stimulation	Stimulation of the left VN, under conditions used to treat epilepsy, does not cause aspiration, though one patient presented laryngeal penetration during stimulation in customized settings
[31]	*N* = 4 (2:2) Patients with intractable epilepsy	Left VN (CI) Neurocybernetic Prosthesis (NCP)	Duration 7–30 s “on” 0.2–5 min “off”; current 0.5–3.25 mA; frequency 15–30 Hz; pulse width 130–500 µsec	-Fiberoptic endoscopic examination of the larynx (without a swallowing protocol) -All patients showed LVF paresis or dysfunction directly after implantation and during stimulation. After adjustment of the settings or during condition “off”, the LVF of two patients recovered. *N* = 1 showed mild aspiration and pyriform sinus pooling during VNS. *N* = 2 showed symptoms of OD (coughing while drinking, handling secretions or gagging), during VNS. *N* = 1 showed improvement of OD after adjustment of VNS	Attachment of the stimulator lead to the VN often results in usually transient vocal fold paresis. Furthermore, VNS itself can also cause vocal fold dysfunction. Clear communication between otolaryngologist and neurologist is essential in controlling potential ARs of the stimulation
[32]	*N* = 3 (3:0) Dysphagic patients with MS and PCT	VN Type of nerve stimulator not specified	Duration 62 s “on”, 60 s “off”; current was eventually 1.25 mA (progression: 0.25 mA increase/week); frequency 10 Hz, pulse width 250 µsec	-50 mL water swallowing speed test -Before start VNS: mean time of 18 s with nine pauses or ‘piecemeal’ deglutition periods (normally 1–2/50 mL of ingested liquid). During the follow-up of 2–3 months, water intake and ‘piecemeal’ deglutition improved by 65% and 78%, respectively, referring to an improved swallowing duration and lower number of piecemeal deglutition	VNS is an alternative in the treatment of advanced MS in which OD is a potentially life threating complication of this disease
[33]	*N* = 9 (4:5) Healthy subjects	ISLN DISA 15E07 stimulator	The pulse generator triggered the DISA stimulator at 0; 500 ms; 1 s; 2 s; 5 s delays after the swallow command. Resulting in four phases: phase A (500 ms): just before onset of the swallow; phase B (1 s): occurring during the swallow; phase C (2 s): occurring within the first 3 s postswallow; phase D (5 s): occurring between 3 and 5 s postswallow	-Signals recorded from the bilateral TA muscles were used to measure laryngeal muscle reflex responses (for airway protection) to electrical stimulation of the ISLN -The more rapid and shorter unilateral responses of TA muscles (R1) to stimulation continued to provide some, albeit reduced, laryngeal protective functions after swallowing, whereas the later contralateral responses (R2) to stimulation were suppressed both in occurrence and amplitude for up to 3 s after swallow (phase C)	The results suggest that the later laryngeal adductor responses are suppressed up to 3 s after the swallow during ISLNS. Residue in the laryngeal vestibule after a swallow, increased the risk for the entry of foreign substances into the airway, when receiving ISLNS
[34]	*N* = 2 (1:1) Hemispheric stroke patients with chronic aspiration	Left RLN Neurocontrol Implantable Receiver-Stimulator	Patient 1: current 1 mA, frequency 42 Hz, pulse width 72–176 µsec Patient 2: current 1.5 mA, frequency 42 Hz, pulse width 20 µsec	-Videofluoroscopy -A significant reduction in aspiration during swallows of thin and thick liquids	The authors concluded that vocal fold pacing via the left RLN is a potentially effective method for the control of aspiration in these two stroke patients
[35]	*N* = 5 (3:2) Patients with various neurologic conditions (MS, CP, stroke) and chronic aspiration pneumonia	RLN Modified Vocare stimulator, Finetech Medical Ltd. Welwyn Garden City, England	Current 1.2 mA; frequency 42 Hz; pulse width, 188–560 μsec	-Videofluoroscopy; chest x-ray; health-related quality of life obtained via patient interview -*N* = 4 showed an overall decrease in aspiration during stimulation, if vocal fold adduction and glottic closure were achieved with RLNS. All patients reported improved health-related quality of life. There was a decrease in the frequency of chronic aspiration pneumonia	Vocal fold pacing via the RLN seems appropriate as treatment for chronic aspiration pneumonia as long as there was glottic seal during the stimulation
[36]	*N* = 14 (9:5) Patients with OSA	HN Type of nerve stimulator not specified	No information on settings	-EAT-10 questionnaire -Only during the first week following the implantation a temporary increase of patient reported dysphagia symptoms was observed	The implantation and use of HNS over five months did not demonstrate any sustained, patient reported changes in OD symptoms

*VN* vagal nerve, *CI* Cyberonics Inc., Houston, Texas, USA., *AR* adverse reaction, *VNS* vagal nerve stimulation, *MS* multiple sclerosis, *PCT* postural cerebellar tremor, *OD* oropharyngeal dysphagia, *LVF* eft vocal fold, *ISLN* internal branch of the superior laryngeal nerve, *TA* hyroarytenoid, *ISLNS* nternal branch of the superior laryngeal nerve stimulation, *RLN* recurrent laryngeal nerve, *CP* cerebral palsy, *RLNS* recurrent laryngeal nerve stimulation, *OSA* obstructive sleep apnea, *HN* hypoglossal nerve, *EAT-10* eating assessment tool-10, *HNS* hypoglossal nerve stimulation

Table 4 provides an overview of the twenty studies that described OD as an AR of CNS. In these studies, the indication for stimulation did not target swallowing function. The following data were extracted from the studies: type of nerve stimulator, stimulation parameters, indication for stimulation, and the number of OD-related ARs. In these studies, ARs such as OD were reported based on patient interview or via Coding Symbols for Thesaurus of Adverse Reaction Terms (COSTART) [28].

**Table 4:** Studies on cranial nerve stimulation for other indications than swallowing, but with OD described as an adverse reaction

Refs	Type of nerve stimulator; stimulus duration, current intensity, pulse frequency, pulse width	Indication for stimulation	OD as AR (*N*)
[37]	VNS (VNS Therapy; CI); ON for 30 s and OFF for 5 min, 20 Hz, LOW (0.25 mA, 130 µsec), MEDIUM (0.5–1.0 mA, 250 µsec), HIGH (1.25–1.5 mA, 250 µsec)	TRD	*N* = 10 (9%) patients in low dose stimulation group, *N* = 17 (15.9%) patients in medium dose stimulation group, *N* = 18 (15.9%) patients in high-dose stimulation group OD was reported as an AR. No further information was provided
[38]	VNS (CI); ON for 30 s and OFF for 5 min, 0.25–3.5 mA, 20 Hz, 500 µsec	TRD	*N* = 25 (21%) patients of the VNS group and *N* = 13 (11%) patients in the Sham group OD was coded using COSTART
[39]	VNS (CI); ON for 7–60 s and OFF for 0.3–180 min, 0.0–2.25 mA, 2–30 Hz, 130–750 µsec	TRD	*N* = 31 (13%) patients during the first quarter of a year; *N* = 19 (8%) patients in the second quarter; *N* = 15 (7%) patients in the third quarter; *N* = 9 (4%) patients during the fourth quarter OD was coded using COSTART
[40]	VNS (CI); ON for 30 s, OFF for 5 min, 0.25–2.0 mA, 10–30 Hz, 250–500 µsec	TRD	Population European (D03) study; after 3 months *N* = 6 (8.5%) patients, 6 months *N* = 0 (0%) patients, 9 months *N* = 0 (0%) patients, 12 months *N* = 2 (3.3%) patients Population USA study; after 3 months *N* = 13 (5.6%) patients, 6 months *N* = 8 (3.6%) patients, 9 months *N* = 7 (3.2%) patients, 12 months *N* = 4 (1.9%) patients OD was coded using COSTART
[41]	VNS (CI); ON for 30 s, OFF for 3–5 min, 0.25–3.0 mA, 20–30 Hz, 250–500 µsec	TRD	*N* = 3 (10%) patients OD was coded using COSTART
[42]	VNS (CI); ON for 30 s, OFF for 3–5 min, 0.25–3.0 mA, 20-30 Hz, 250–500 µsec	TRD	*N* = 8 (13%) patients. Onset of OD after a mean duration of 11.3 days. OD spontaneously resolved in *N* = 7 (87.5%) patients OD was coded using COSTART
[50]	VNS; mean values (ON for 21.6 s and OFF for 1.1 min, 1.3 mA, 25 Hz, 220 µsec)	TRD and bipolar disorder	*N* = 1 (6.7%) OD was reported as AR. No further information was provided
[43]	VNS (CI); ON for 7 s or 30 s, OFF for 12–30 s or 3–5 min, 0.25–2.0 mA, 30 Hz, 500 µsec	Intractable epilepsy	*N* = 2 (12.5%) patients showed repeated coughing, swallowing difficulties, and significant increase of aspiration when stimulator was turned ON OD reported via patient interview and videoradiography during barium swallow
[44]	VNS (CI)	Partial-onset seizures refractory to medical therapy	*N* = 4 (8.7%) patients OD was reported using an AR questionnaire comprising six questions aimed to determine the effect of VNS on swallowing function. Reprogramming the device seemed to have little effect on swallowing
[45]	VNS; ON for 7–30 s, OFF for 18 s-5 min, 0.25–2.0 mA, 20 Hz	Intractable focal epilepsy	*N* = 12 (80%) patients reported cough, hoarseness, and mild OD OD was reported as AR. No further information was provided
[46]	VNS	Intractable pediatric epilepsy	*N* = 3 (4.8%) patients OD was reported as AR. OD was managed by reprogramming the device or a temporary switch-off of the device using a magnet. No further information was provided
[48]	VNS; ON for 7–30 s, OFF for 30 s-5 min, 0.25–3.0 mA, 20–30 Hz 250–500 µsec	Drug-resistant seizures	*N* = 3 (4%) patients OD was reported as AR. No further information was provided
[49]	VNS (CI); ON for 30 s, OFF for 5 min, 0.25–2.0 mA, 30 Hz, 500 µsec	Intractable Epilepsy	*N* = 2 (7.4%) patients OD was reported as AR during VNS. No further information was provided
[51]	VNS (CI); ON for 30 s and OFF for 5 min, 0.25 mA, 20 Hz, 250 µsec	Medically refractory multifocal or generalized epilepsy	*N* = 2 (2.8%) patients reported intermittent OD OD was reported as AR. OD resolved by adjusting the stimulation parameters. No further information was provided
[52]	VNS (CI); mean values (ON for 29 s and OFF for 4 min, 2.04 mA, 25 Hz, 307 µsec)	Intractable epilepsy	*N* = 1 (1%) patient suffered from transient OD OD was reported as AR. No further information was provided
[53]	VNS (CI); ON for 14–30 s and OFF for 1.1–5 min, 0.75–2.5 mA, 20–30 Hz, 500 µsec	Drug-resistant epilepsy	*N* = 1 (1.8%) patient reported OD and vomiting OD was reported as AR. No further information was provided
[16]	VNS	Medically intractable epilepsy	*N* = 1 (1.4%) patient OD was reported as AR. Reprogramming the device did not improve OD. No further information was provided
[54]	VNS; ON for 7–30 s and OFF for 30 s-5 min, 0.25–2.25 mA, 20–30 Hz, 250–500 µsec	Refractory epilepsy	*N* = unknown. OD is, among hoarseness and coughing, the most common AR No further information was provided
[47]	VNS (CI); ON for 30 s, OFF for 5 min, 0.5–2.5 mA, 20 Hz, 250 µsec	Treatment-resistant fibromyalgia	*N* = 2 (14%) patients OD was reported as AR. No further information was provided
[36]	VNS (Model 304; CI; 500 ms, 0.8 mA, 30 Hz, 100 µsec	Chronic ischemic stroke	*N* = 1 (11%) patient showed transient OD and left vocal fold palsy after device implantation. *N* = 1 (11%) patient reported mild OD in the evening after therapy sessions OD was reported via patient interview. Other causes for vocal fold palsy than stimulation were excluded by a computed tomographic scan

*OD* oropharyngeal dysphagia, *AR* adverse reaction, *VNS* vagal nerve stimulation, *CI* Cyberonics Inc., Houston, Texas, USA, *TRD* treatment-resistant depression, *COSTART* Coding Symbols for Thesaurus of Adverse Reaction Terms

### 3.2 Summary of Studies

The studies in Table 3 were presented according to the cranial nerve number stimulated (from low-to-high): VN (four studies), ISLN (one study), RLN (two studies), and HN (one study).

### Vagal Nerve Stimulation

Lundgren et al. primarily studied swallowing in seven children, treated for intractable epilepsy with a VN stimulator using videoradiography with barium swallowing [29]. Three stimulation conditions were compared to observe the effects of VNS on swallowing (“MAX”; “in use”; “off”). During almost continuous VNS (“MAX”) an increased penetration-aspiration score was observed in two of the seven children. In one patient, this increased score during “MAX” was observed compared to the “in use” setting and in the other patient it was observed during “MAX” compared to the “off” setting. However, no significant difference in penetration-aspiration score was found between the “in use” versus “off” condition in all children.

Schallert et al. studied eight children with pharmacoresistant epilepsy using a left-sided-VN stimulator to determine whether stimulation could affect swallowing [30]. They concluded that stimulation of the left VN, under the conditions used to treat epilepsy, did not cause aspiration during barium swallow videoradiography, although one patient showed laryngeal penetration during “therapeutic stimulation” (condition “on”) versus no laryngeal penetration during no stimulation (condition “off”).

Zalvan et al. retrospectively reported ARs of VNS on swallowing in four patients with intractable epilepsy in a case series study [31]. Swallowing was evaluated using patient interview and fiberoptic endoscopic evaluation of the laryngeal function. Symptoms of OD, such as gagging or coughing while drinking or feeding difficulties during stimulation (condition “on”), were reported by three of the four subjects. Furthermore, the authors reported that these OD-related symptoms persisted in one subject despite switching off the stimulator (condition “off”). After adjustment of the VNS parameters, two subjects did not show OD-related symptoms anymore.

Marrosu et al. investigated the effect of VNS on swallowing in three males affected by multiple sclerosis (MS) presenting postural cerebellar tremor (PCT) and OD [32]. Following VNS OD for thin liquid measured using a swallowing speed test and the PCT improved in all subjects during the follow-up period of two to three months. Difficulties in swallowing solids failed to improve, although specific information on swallowing outcome variables was not reported.

### Internal Branch of the Superior Laryngeal Nerve Stimulation

Central nervous system suppression of laryngeal adductor responses during swallowing was studied in nine healthy subjects by Barkmeier et al. [33]. They studied the frequency and amplitude of laryngeal adductor responses in the thyroarytenoid (TA) muscle such as the rapid and shorter ipsilateral (R1) responses and later contralateral (R2) responses. The authors used bipolar needle electrodes during electrical stimulation of the ISLN in different phases of volitional swallowing. The results demonstrated a suppression of laryngeal sensorimotor R2 responses up to three seconds following a swallow command. The R1 response frequency, however, was not affected during all phases of swallowing. These results suggested that stimulation of the ISLN is suppressing the R2 laryngeal protective function (adductor responses), putting people at risk for aspiration. However, the R1 rapid protective sensorimotor response remained intact and could still be triggered during bolus entry into the laryngeal vestibule and penetration up to the level of the vocal folds evoking a cough reflex.

### Recurrent Laryngeal Nerve Stimulation

Broniatowski et al. described the effect of RLN stimulation (RLNS) on aspiration in two stroke patients with a tracheostomy and chronic aspiration [34]. There was a significant reduction in aspiration during videofluoroscopy in these two patients during thin and thick liquid swallows under RLNS. Swallows of puree consistency did not improve during RLNS. The authors concluded that swallowing of thin and thick liquid consistencies was safe as a result of RLNS.

Subsequently, Broniatowski et al. studied the effect of RLNS on aspiration during videofluoroscopy in three additional patients extending their sample size to five patients (including the two patients of their previous study) [35]. The effect of RLNS on health-related quality of life, measured with a patient interview and pneumonia rates was reported in the study. Patients were suffering from stroke, MS, or cerebral palsy. In four of the five patients, the frequency of aspiration decreased using RLNS and their health-related quality of life improved. One stroke patient did not experience any beneficial effect of RLNS on health-related quality of life or on pneumonia rate. The authors concluded that RLNS is a potentially effective method for the reduction of aspiration and that RLNS might have an added value in the prevention of chronic aspiration pneumonia.

### Hypoglossal Nerve Stimulation

Bowen et al. described the effect of HNS on swallowing in patients treated for obstructive sleep apnea (OSA) [36]. During the first week following the implantation of the HN stimulator, a temporary increase of OD symptoms, measured with the EAT-10 questionnaire, was observed, which normalized during the next six months. This might indicate that the transient OD-related symptoms were probably due to postoperative wound healing conditions at the implantation site.

Table 4 presents 20 studies where signs of OD such as impaired swallowing safety (aspiration) or impaired swallowing efficiency (pooling/residue) were described as an AR of CNS. In the majority of these studies, ARs were identified using COSTART, swallow-related questionnaires and/or physical examination. However, in the vast majority of these studies ARs were reported without any information on the applied AR protocol or any other protocol for data collection. In nearly half of these studies, OD was not specified in terms of onset, severity, duration, or dependency of stimulation parameters. In these studies, the frequency of OD signs and symptoms ranged from 1 to 80%.

## Discussion

Despite the fact that the number of indications for CNS has increased in recent years, the number of studies on the effect of this stimulation on swallowing has remained very limited. The aim of this systematic literature review was to present an overview of the studies on the effect of CNS on swallowing and to determine the level of evidence of these studies to guide the development of future research on new treatment strategies for OD using CNS. The systematic search we conducted for this review generated a limited number of articles on the effect of CNS on swallowing [29–36]. Clear conclusions about the evidence could not be drawn as the majority of the included studies were case studies or case series and the overall sample size was very small. Study size varied from 2 to 14 subjects. Four studies analyzed a population of less than five subjects [31, 32, 34, 35]. None of the studies described statistical analyses. The majority of the studies did not report the diagnostic protocol used to assess the swallowing physiology. The studies were considerably heterogeneous regarding patient populations and outcome parameters. The study populations consisted of children and adults with epilepsy, healthy subjects, patients with various neurologic conditions (MS, CP, and stroke), and patients with OSA. No information was reported on any long-term effects of CNS on swallowing. It can be concluded that all included studies of the present review did not clearly describe the methodological study protocol making replication of the studies impossible.

The effect of direct electrical stimulation of the TN, FN, and GN on swallowing, either as an AR during stimulation for other non-swallow indications, has not been reported in the literature yet.

Meanwhile, the effects of VNS and HNS on swallowing were primarily reported as ARs [17, 37–55]. For example, in the treatment of depression, VNS seems to induce OD in up to 10% of the patients according to the systematic review of Martin et al. [56].

The most frequently used nerve for CNS is the VN. VNS has shown to be a minimally invasive procedure in which the stimulator is generally well tolerated compared to other neurosurgical interventions such as deep brain stimulation, motor cortex stimulation, responsive neurostimulation, or spinal cord stimulation [10, 11, 13–20, 37–46, 48–58]. The VNS device consists of a pulse generator which is placed subcutaneously in the upper chest below the patient’s clavicle and bipolar electrodes are tunneled up to the patient’s neck and wrapped around the left VN, above the level of the omohyoid muscle in the neurovascular sheet. Although the therapeutic effects of VNS were extensively studied, the exact therapeutic mechanisms underlying neurostimulation remain unclear [58]. It is known that VNS activates neurons in the basal forebrain and locus coeruleus. This activation evokes a release of acetylcholine and norepinephrine facilitating reorganization of cortical networks and enhancement of neural plasticity [59, 60]. It has been suggested that VNS in conjunction with muscle movement might improve task-specific plasticity in the motor cortex of rats [61]. Subsequent studies on human subjects showed that VNS in conjunction with rehabilitative muscle training drives large-scale synaptic reorganization in motor control networks following stroke and spinal cord injury, and provides an enduring rehabilitation [62, 63]. Current studies are investigating optimal settings for VNS in rats to identify paradigms that maximize neuroplasticity [64]. The question arises whether VNS could contribute to an improvement of neuroplasticity in case of OD due to neurological disorders?

Usually the VN is stimulated for drug-resistant epilepsy and depression [58]. The potential use of VNS for other indications, such as essential tremor, cognitive deficits in Alzheimer’s disease, anxiety disorders, and bulimia has been reported in several studies [65]. Furthermore, the effect of anti-epilepsy VNS on swallowing was described in several studies [29–31]. These studies reported increased signs of OD including aspiration as an AR of VNS. Aspiration disappeared in the majority of patients following adjustment of the stimulation parameters. One study described VNS as a primary treatment for OD in patients with MS showing a beneficial effect of VNS on OD complaints [32]. Currently, the level of evidence of studies on VNS is too poor to support VNS as a treatment for OD.

The HN comes in second place as most frequently stimulated cranial nerve and is usually stimulated as a treatment for OSA [58]. Only one single study described the effect of HNS for OSA on swallowing in non-dysphagic patients. A short-term self-limiting patient reported AR on swallowing occurred immediately following surgical implantation. HNS did not have any long-term ARs on swallowing in this study. Yet, it is known that damage of this nerve usually results in dysarthria and problems in the preparatory oral phase and the oral phase of swallowing [66]. An interesting hypothesis arises if we look at the study by Hadley et al. on the effect of HNS in anesthetized canines. The HNS resulted in an increased hyolaryngeal elevation, which plays an important role in airway protection during swallowing [67]. The remaining question is whether HNS can contribute to swallow safety in patients presenting aspiration.

The current systematic review only identified a single study describing the effects of ISLN stimulation (ISLNS) on volitional swallowing in healthy subjects [33]. The authors concluded that ISLNS resulted in an increased risk of aspiration due to suppression of the protective laryngeal adductor responses. Even isolated peripheral ISLN dysfunction without any lesions in the airway or central nervous system can cause an increased aspiration risk [33]. Relevant additional information about the role of the ISLN in swallowing has been described in the study by Jafari et al. They reported that bilateral anesthetic blockage of the ISLN with 0.5% bupivacaine has a profound effect on swallowing in all twenty-one healthy subjects [68]. These healthy subjects showed an increased effort to initiate swallowing and 25% of them showed penetration or aspiration during videofluoroscopy with liquid barium. Further studies on the effect of ISLNS in dysphagic patients are needed.

Two studies evaluated the effect of RLNS on OD in stroke, MS, and CP patients suffering from chronic aspiration pneumonia [34, 35]. The authors concluded that RLNS seems to improve the glottal seal which might be a potentially effective method to prevent aspiration in these patients. But here too, due to methodological shortcomings the scientific evidence of these studies is so weak that RLNS cannot be recommended as a treatment for OD. Swallowing is a very complicated neurogenic activity that is elicited through a collaboration between different integrated neural pathways in the central and peripheral nervous systems [8]. Miller et al. described that stimulation of specific cranial nerve nuclei in the brainstem did not result in a pharyngeal swallow [69]. However, it remains an interesting question whether stimulation of cranial nerves in their course or at the level of their nuclei in the brainstem can bring about a change that may in the long term be relevant in the context of treatment of certain OD phenotypes. Overall, the research question whether there is a beneficial effect of CNS on swallowing remains unanswered.

### Limitations of the Review

The present review has some limitations with respect to the search strategy and data analysis. The systematic search generated a low number of articles on the effect of CNS on swallowing in human subjects. One reason for this low number may be the inconsistent terminology used in this research topic; as a result, it is possible that some eligible studies were missed despite the extended free-text search (Table 1). The systematic search syntax and the analysis of the level of evidence were carried out according to the PRISMA statement. However, the study designs were of such poor methodological quality that a subsequent meta-analysis or qualitative analysis using a validated critical appraisal tool could not be performed. The steps following the analysis of the level of evidence have to be classified under narrative or traditional literature review with a comprehensive, critical, and objective analysis of the current knowledge on this topic. The gray literature was not included because it generally lacks strict bibliographic control, meaning that basic information such as author, publication date, or publishing body may not be easily discerned. Publication bias cannot be ruled out as it is likely that unpublished studies did not find any effects of CNS on swallowing or resulted in severe ARs.

## Conclusion

These preliminary data suggest that specific well-indicated CNS might be effective in reducing OD symptoms in selective patient groups. But it is far too early for conclusive statements on this topic. The reviewers found heterogeneous outcomes and serious methodological.

limitations, which prevented them from pooling data to identify trends that would assist in designing best clinical practice protocols for OD using CNS. However, these first pioneer studies are very important because they confirm the feasibility of CNS as possible treatment option for OD. To date, it is not known whether CNS for OD could have negative effects. Therefore, the risk/benefit ratio should also be included in future studies. In conclusion, the results of these studies are encouraging for future research on CNS for OD. However, randomized, double-blind, sham-controlled clinical trials with adequate sample size are necessary.
